# Identification and phased de novo mutation of the EPS8L2 gene in a patient with progressive hearing loss: A case report

**DOI:** 10.1097/MD.0000000000046930

**Published:** 2026-01-23

**Authors:** Hanxiao Gan, Yu Lu, Shenhong Qu, Lianli Lu, Huilan Xu, Mingyin Fan, Jie Tang, Jinying Mo, Ruichun Chen, Fengzhu Tang

**Affiliations:** a Department of Otolaryngology and Head and Neck, The People’s Hospital of Guangxi Zhuang Autonomous Region, Nanning, Guangxi, China; b Graduate School of Guangxi Medical University, Nanning, China; c Department of Oto-Rhino-Laryngology, West China Hospital of Sichuan University, Chengdu, China; d Institute of Rare Diseases, West China Hospital of Sichuan University, Chengdu, China.

**Keywords:** epidermal growth factor receptor pathway substrate 8 L2 (EPS8L2), hearing loss, new disease-causing variant, progressive deafness, whole genome sequencing

## Abstract

Pathogenic variants in the EPS8L2 gene are known to underlie nonsyndromic progressive hearing loss. In this study, we enrolled a 39-year-old male patient with deafness. We collected detailed clinical characteristics and results of auxiliary examinations, followed by whole-genome sequencing to analyze 189 genes commonly associated with hereditary deafness. Sanger sequencing and T/A cloning were employed to validate candidate variants, leading to the identification of 2 frameshift mutations in the EPS8L2 gene in a compound heterozygous state and in trans: c.357_361dupGGTGC (p.Gln121Argfs67) and c.1317dupG (p.Leu440Alafs63). Notably, the c.357_361dupGGTGC variant was a de novo mutation in the patient, whereas c.1317dupG was inherited from his mother. Both novel frameshift mutations are predicted to result in severely truncated proteins, and the affected amino acid residues are highly conserved across diverse species. To date, only 2 cases of EPS8L2-associated hearing loss caused by homozygous pathogenic variants have been reported in a single family. Herein, we describe the first case of nonsyndromic autosomal recessive hearing loss 106 (DFNB106) harboring compound heterozygous pathogenic variants in EPS8L2. These findings expand the mutational spectrum of EPS8L2, provide critical insights for genetic diagnosis, genetic counseling, and prognosis assessment of progressive hearing loss caused by EPS8L2 variants, and further offer a clinical reference for identifying de novo compound heterozygous mutations in autosomal recessive nonsyndromic hearing loss (DFNB106) cases, aiding in more accurate etiological identification and targeted management of similar progressive hearing loss patients.

## 1. Introduction

Deafness is the most common sensory impairment in humans and not only affects individuals, but also places a heavy burden on families and society. In 2021, the World Health Organization (WHO) estimated that 1.5 billion people worldwide have hearing loss, and it is expected that the number of deaf people will increase globally by 2050.^[1]^ According to the Second China Disability Sample Survey, hearing-impaired people account for approximately 32.9% of the total number of people with disabilities in China and approximately 1.54% of the national population.^[2]^ Genetically related deafness can be classified into nonsyndromic hearing loss (NSHL) and syndromic hearing loss according to whether the patients suffer from other systemic diseases. Among these cases, NSHL accounts for approximately 70% of cases.^[3]^ On the basis of the function and expression pattern of the inner ear, nonsyndromic genes can be broadly classified into 4 main categories: those involved in hair bundle morphogenesis, those that play a role in ion homeostasis, those that participate in extracellular matrix composition and those that act as transcription factors.^[4]^ Among them, EPS8L2 is one of the genes involved in hair bundle morphogenesis.

NSHL can be divided into prelingual and postlingual deafness. Among these, prelingual deafness has a high prevalence and a greater impact on children’s lives and learning than does postlingual deafness; however, postlingual deafness has not received much clinical attention because its prevalence and impact are not as profound as those of prelingual deafness. To date, only a few causative genes for postlingual deafness have been reported. Autosomal recessive non-syndromic hearing loss (ARNSHL), which is genetically highly heterogeneous, is the most common form of inherited deafness, and at least 87 genes have been associated with autosomal recessive nonsyndromic inherited deafness (https://hereditaryhearingloss.org; accessed September 2024). Approximately 25% of the identified DFNB genes are associated with progressive deafness.^[5]^ The EPS8L2 gene is similar to the genes that have been reported to be associated with DFNB in progressive ARNSHL in humans (*SLC26A4*,^[6,7]^ *MYO3A*,^[8]^ *TPRN*,^[9,10]^ *TMPRSS3*,^[11,12]^ *GRXCR2*,^[13]^ *CLIC5,*^[14]^ *TMC1*,^[15]^ *GRXCR1*,^[16]^ and others), and patients may present with a progressive post-speech hearing loss phenotype.

In the mammalian cochlea, the hair bundle is typically composed of 3 rows of stereocilia. Hair cells perceive the mechanical stimulus of sound through their apical cilia. The cytoskeletal core of stereocilia is composed of tightly packed actin filaments, as evidenced by findings from previous studies.^[17,18]^ The length of stereocilia is regulated to ensure the characteristic stepped structure of each bundle. One of these genes, EPS8L2 (OMIM617637), encodes a novel actin filament capping protein that plays a role in the long-term maintenance of the stepped structure of hair bundles and the mechanosensory function of auditory hair bundles. The gene is located at 11p15.5, comprises 22 exons and 3030 base pairs, and encodes a 715 amino acid precursor protein containing a phosphotyrosine-interacting structural domain (PID), an SRC homology 3 (SH3) structural domain, and an effector region including SAM/PNT. EPS8L2 is thought to be a DFNB gene associated with human progressive ARNSHL.

Here, we report a case of a male who experienced mild-to-severe sensorineural deafness changes. The novel compound heterozygous mutations c.357_361dupGGTGC (p.Gln121Argfs*67) and c.1317dupG (p.Leu440Alafs*63) were detected in the EPS8L2 gene. The former variant was identified as de novo and phased the biallelic genotype by monoclonal amplification. This compound mutation results in amino acid shifts and premature truncation of highly conserved structural domains, leading to damage to the mutant protein.

## 2. Materials and methods

### 2.1. Subjects and clinical examinations

We recruited a 39-year-old male and conducted a detailed enquiry of family members, including age of onset, disease progression, disease history, family history of deafness, and history of trauma. Physical, laboratory and imaging examinations were also performed. Peripheral blood was drawn from the patient and his family members after informed consent was obtained. The study of this family line was approved by the Ethics Committee of the People’s Hospital of Guangxi Zhuang Autonomous Region (Scientific Research Guangxi Science and Technology-2017-29).

### 2.2. High-throughput sequencing

The DNBSEQ-T7 sequencing platform was used to sequence DNA extracted from the blood of the proband. The following quality control filters for mutations were applied: read depth > 6, genotype qualities > 20, and allele frequency > 10%. The VarSeq software cloud platform was used to integrate gene variant databases (including the dbSNP, ClinVar, HGMD pro, GnomAD, and OMIM databases) and filter mutation sites with minor allele frequency ≥ 0.005 and mutations annotated as benign mutations. Mutations in the coding and shear site regions of deafness genes were screened, and candidate mutations were analyzed for pathogenicity. The American College of Medical Genetics and Genomics (ACMG) standards and guidelines were used to interpret the pathogenicity of the gene variants. The reference transcript for the gene mentioned in this study is EPS8L2: NM_022772.4.

### 2.3. EPS8L2 gene amplification and TA cloning

PCR amplification of an EPS8L2 gene fragment in the patient’s genomic DNA sample was performed with the following primers: P1 (forward primer), 5′-GAGTCGTGTCCGCGCGATGTAC-3′; P2 (reverse primer), 5′-GAGCTCTGGTGAGGAGTGGGTGTTG-3′. The reaction system included 1 μL of the DNA template, 1 μL of the upstream and downstream primers, 0.25 μL of TakaRa Taq HS, 5 μL of 10 PCR Buffer, 4 μL of the dNTP mixture, and ddH2O to 25 μL. The PCR conditions were as follows: 94°C for 3 minutes, followed by 35 cycles of 94°C for 30 seconds, 62°C for 15 seconds, and 72°C for 30 seconds, ending with a final extension at 72°C for 5 minutes. Then, 1.2% agarose gel electrophoresis was performed, and the highlighted region of the band was cut and collected in a 1.5 mL centrifuge tube. The target fragments were recovered according to the instructions provided in the kit. After the concentration and purity were tested, the vector pMD19-T was transformed into DH5α competent cells, monoclonal plaques were picked for PCR, and positive clones were picked and sent to a company for sequencing.

### 2.4. Mutation prediction

Three-dimensional structural molecular modeling of the wild type and mutant EPS8L2 was performed by means of AlphaFold and PyMOL.

## 3. Results

### 3.1. Hearing, physical examination and imaging results

**Figure 1.**
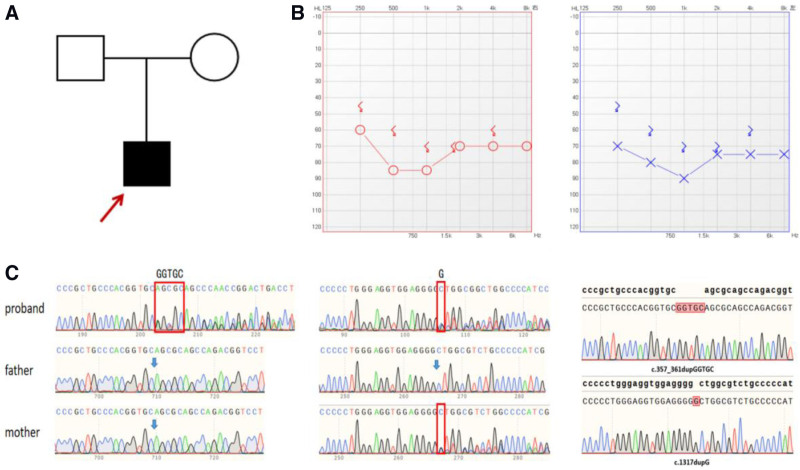
Pedigree and genetic features of the family. (A) A pedigree map with 3 generations was shown with proband highlighted by the arrow. (B) Pure tone audiometry showed profound bilateral hearing loss in proband. Blue: left; red: right. (C) Sanger sequencing showed a genotype of 3 family members. Blue arrows represent the location of the repeats. (D) Single clone sequencing results: alignment of the template strand and PCR products.

The patient was a 39-year-old male who was born to unrelated normal-hearing parents (Fig. 1A). He was delivered at the hospital at full term. His speech, growth and intellectual development were normal, and he denied having ototoxic medication, ear trauma, ear surgery, prolonged noise exposure, early pregnancy infection, cardiovascular disease and diabetes. According to his recollection, he was aware of his hearing loss when he was 16 years old and did not undergo any special treatment or therapy, and his hearing test during the physical examination for the university entrance exam indicated that he had mild hearing loss in both ears. His hearing deteriorated over time during his college years, and at age 27, he began wearing a unilateral (right) hearing aid to help with sound perception. Currently, his pure tone audiometry is well above the threshold (Fig. 1B). Cranial computed tomography and magnetic resonance imaging were unremarkable. The patient’s tympanometry curve was type A, suggesting normal middle ear function.

### 3.2. Whole-genome sequencing

Whole-genome sequencing of the proband revealed 2 heterozygous mutations (NM_022772.4:c.357_361dupGGTGC (p.Gln121Argfs*67) and NM_022772.4:c.1317dupG (p.Leu440Alafs*63)) in the EPS8L2 gene. Neither has been reported in the literature or in the ClinVar, PubMed or HGMD databases. These mutations lead to premature termination of amino acid transcription, produce severely truncated proteins, and may have strong effects on protein structure and function. These proteins may activate the NMD pathway to underlie the pathogenesis of hearing loss through a loss-of-function mechanism.^[5]^ Additional variants in other genes were also detected during sequencing. We annotated these variants and assessed their potential pathogenicity by integrating their positions, types, frequencies, and known associations with pathogenicity, and EPS8L2 was identified as the causative gene through this screening process. Sanger-based sequencing confirmed that the latter variant (c.1317dupG) originated from the mother and that both alleles in the father were wild type (Fig. 1C), suggesting that the former p.Gln121Argfs*67 is a de novo variant.

### 3.3. TA cloning

To further clarify whether the 2 heterozygous variants in the patient’s EPS8L2 gene were in cis or in trans, a segment of the EPS8L2 gene containing these 2 positions from the patient’s DNA sample was amplified via PCR and sequenced via TA cloning. The sequencing results revealed that the 2 variants were located on different single DNA alleles, indicating that the shifted variant p.Leu440Alafs*63 occurred in a single allele of the patient’s maternal DNA and that p.Gln121Argfs*67 was a de novo variant. At this point, it can be concluded that the variants c.357_361dupGGTGC (p.Gln121Argfs*67) and c.1317dupG (p.Leu440Alafs*63) in the patient are in trans (Fig. 1D), and there is a compound heterozygous variant in the EPS8L2 gene in the patient.

### 3.4. Mutation prediction and multiple sequence comparison

**Figure 2.**
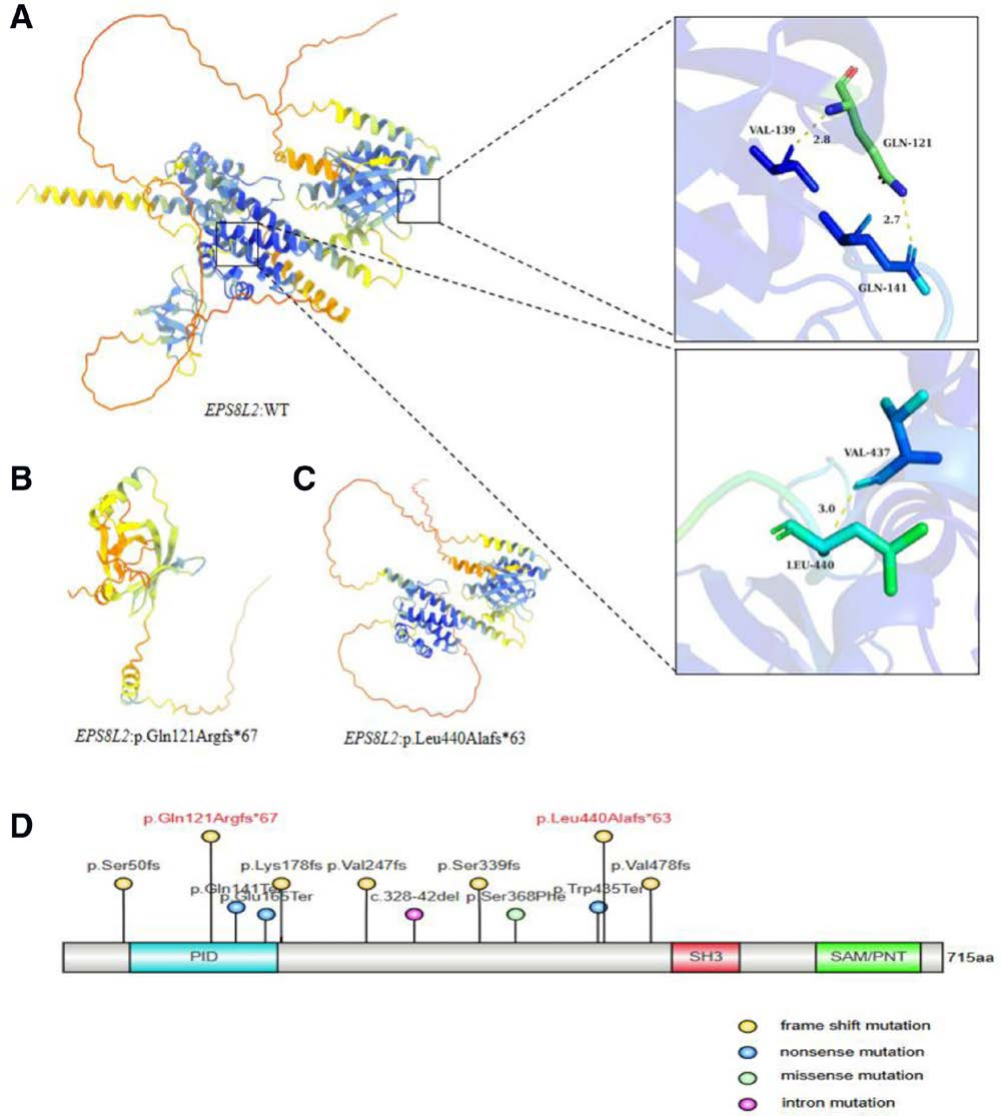
The prediction model of the mutant protein and EPS8L2 protein structure. (A) WT EPS8L2 protein shows Gln in green at 121 and Leu in sky blue at 440; (B, C) 3 D protein modeling showed that p.Gln121Argfs*67 and p.Leu440Alafs*63 cause the appearance of frameshift and early stop codons, resulting in a severely truncated EPS8L2 protein that impairs its normal function. (D) A Schematic diagram of the EPS8L2 protein structure. Red color indicates the pathogenic locus found in this study.

3D ribbon model in silico prediction was performed, and the c.357_361dupGGTGC (p.Gln121Argfs*67) and c.1317dupG (p.Leu440Alafs*63) mutations were predicted to cause frameshift and emergence of an early stop codon, resulting in the production of a severely truncated protein (Fig. 2A–C). These results indicate that the c.357_361dupGGTGC and c.1317dupG mutations likely affected the structure and function of the protein. These findings suggest that compound heterozygosity for both mutations may be responsible for severe hearing impairment in preexisting individuals.

## 4. Discussion

In this study, we report a patient with postlingual progressive sensorineural deafness, in which a compound heterozygous mutation of c.357_361dupGGTGC and c.1317dupG in the EPS8L2 gene was detected by whole-genome sequencing. c.357_361dupGGTGC (p.Gln121Argfs*67) and c.1317dupG (p. Leu440Alafs*63) result in 5 bp and 1 bp repeats, respectively, in the exons of the EPS8L2 gene, both of which are predicted to result in translocations leading to premature termination of translation. The patient’s phenotype is also consistent with the typical features of autosomal recessive nonsyndromic progressive deafness caused by the EPS8L2 gene. In our study, Sanger sequencing revealed that the c.357_361dupGGTGC (p.Gln121Argfs*67) variant was not inherited from either parent, suggesting that it and the c.1317dupG variant could originate from 2 different chromosomes in the patient or from the same chromosome. As an autosomal recessive disorder, its development generally requires the presence of deleterious mutations in both chromosomes. Therefore, the laboratory used TA clonal sequencing to further analyze the origin of the mutation and confirmed that c.357_361dupGGTGC is a de novo mutation and that compound heterozygosity is present in the patient in a trans relationship. This provides a basis for assessing the pathogenicity of the variant according to the ACMG/AMP guidelines.

According to the ACMG guidelines, the evidence for the c.357_361dupGGTGC mutation is “PVS1_Strong + PM2 + PS2_Supporting,” and the evidence for the c.1317dupG mutation is “PVS1_Strong + PM2 + PM3,” which are considered pathogenic mutations. Therefore, the compound heterozygous mutation c.357_361dupGGTGC and c.1317dupG in the EPS8L2 gene is pathogenic in this patient. Out of the documented cases involving EPS8L2 variants, approximately 4 reported postlingual onset of symptoms (Table 1).

**Table 1:** Cases involving the EPS8L2 variant are reported in the literature.

Nucleotide variation	Amino acid changes	Variants type	Phenotype	Population (Hom or Het)	Onset pre- post-lingual	References
c.357_361dupGGTGC	p.Gln121Argfs*67	Frameshift (premature stop)	Moderate to profound postlingual progressive-delayed SNHL	Chinese (Het)	Post-lingual	This study
c.1317dupG	p.Leu440Alafs*63	Frameshift (premature stop)	Moderate to profound postlingual progressive-delayed SNHL	Chinese (Het)	Post-lingual	This study
c.148insGGACA	p.Ser50Trpfs*34	Frameshift (premature stop)	Profound SNHL	Palestinian (Hom)	Pre-lingual	Abu Rayyan et al ^[19]^
c.1430dup	p.Val478Serfs*25	Frameshift (premature stop)	Progressive’ found SNHL	Palestinian (Hom)	Post-lingual	Abu Rayyan et al ^[19]^
c.1340G > A	p.Trp435Ter	Nonsense	SNHL	Chinese (Hom)	NA	Wu et al ^[20]^
c.737delC	p. Ala246Alafs*6	Frameshift (premature stop)	Moderate SNHL	Palestinian (Hom)	Post-lingual	Wang et al^[21]^
c.1014delC	p.Ser339Alafs*15	Frameshift (premature stop)	Moderate to severe postlingual progressive-delayed SNHL	Algerian (Hom)	Post-lingual	Dahmani et al^[5]^

Several unique loss-of-function variants of the EPS8L2 gene have been reported in human families, but the relationship between genotype and phenotype has not been fully characterized. In 2015, there was a report of an Algerian consanguineous family in which c.1014delC in a purely heterozygous state was detected in 2 children; however, these children were found to have hearing loss at age 4 years with no other specific phenotype. In the heterozygous status of the unaffected parents and an unaffected sister, the genes were all purely heterozygous variants segregating with the disease in other family members. In 2017, the EPS8L2 mutation was identified in a consanguineous marriage in a Pakistani NSHL family.^[21]^ Two affected children were found to be homozygous for the mutation, with the c.737delC variant, while the unaffected parent was also heterozygous but with an unaffected sister. One case of EPS8L2 c.1340G > A^[20]^ was confirmed via NGS screening in the 2019 Taiwan, China cohort. Two families ina large cohort of Palestinian deafness genetic studies revealed that the EPS8L2 c.148insGGACA and EPS8L2 c.1430 dup^[19]^ mutations were homozygous, the former producing severe hearing loss and the latter leading to progressive hearing loss. At present, fewer EPS8L2 mutations have been reported in deaf patients; in the ClinVar, HGMD and DVD databases, nonsyndromic deafness-related pathogenic and possibly pathogenic mutations, including 5 frameshift mutations, 4 nonsense mutations, and 1 intron mutation, are present in a total of 10 kinds of deafness, and the most common ones include frameshift mutations and nonsense mutations. The included mutation sites are more consistent with the location of the sites found in this study and may lead to defective gene function by affecting loss of function (Fig. 2D), which further validates the pathogenicity of the mutations reported in this study.

The hearing characteristics of the patients in this study were similar to those previously reported in human individuals and animal models, all of which showed progressive NSHL. However, the age of onset was different in human individuals, with autosomal recessive progressive hearing loss with childhood onset reported previously and progressive hearing loss in adolescence in this case, perhaps in relation to the specific genetic pleiotropy and complex mutant genotype–phenotype associations of compound heterozygous mutations in the EPS8L2 gene. Patients carrying different mutations may exhibit different clinical phenotypes. Therefore, identifying novel mutations and deepening our understanding of the relationship between the effects of these mutations and clinical phenotypes will aid in the diagnosis and treatment of genetic mutations.

EPS8L2, a member of the EPS8-like protein family, has the capacity to trigger Rac-GEF activity and to substitute for eps8.^[22]^ EPS8L2 is the member with the highest structural similarity to EPS8, suggesting the existence of functional redundancy within the pathway. The distribution of Eps8L2 overlaps with that of Eps8,^[23,24]^ and both are expressed at the tips of stereocilia. However, there is a subtle difference in their expression patterns. The spatial and temporal expression patterns of the 2 proteins differ. Eps8 is more abundant in the highest rows of stereocilia, whereas EPS8L2 is more abundant in the middle and lowest rows of stereocilia but almost undetectable in the highest rows. Eps8 is located at the tip of stereocilia,^[25]^ where it regulates the normal growth of the tufts of hairs.^[23,24]^ Eps8L2 is also located predominantly at the tip of stereocilia but is involved mainly in the stabilization of hair bundles after their formation. The difference in expression between the 2 proteins may be one of the reasons for the difference in function, but the 2 proteins together contribute to the normal development and maintenance of the hair bundle structure. EPS8 knockout mice exhibit severe congenital deafness,^[23,24]^ whereas EPS8L2 knockout mice exhibit progressive hearing loss with progressive deterioration of inner ear hair bundles.^[26]^ The progressive hearing loss phenotype observed in EPS8L2 knockout mice closely resembles the hearing loss and deafness observed in Rhodesian Ridgebacks, which manifests during childhood and early adulthood. This evidence suggests that EPS8L2 plays a crucial role in maintaining mature hair cells in the inner ear.^[5,26]^

Most of the previously reported deaf families were consanguineous, whereas the Guangxi families identified in this study were nonconsanguineous; however, the patients were found to be compound heterozygous for very rare mutations. The mutation frequency and hotspot mutations are race-specific and region specific, and the mutation spectrum and dominant mutations of deafness genes are different in different ethnic groups and regions. The mutation loci found in this study were not detected in the databases of various populations, and further studies are needed to determine whether they are locally specific mutations in Guangxi.

## 5. Conclusion

Two novel frameshift mutations in the EPS8L2 gene were detected in healthy nonconsanguineous Chinese parents with progressive hearing loss. De novo variant was rarely reported in autosomal recessive diseases, the in trans genotype in this study was identified by monoclonal amplification. The mechanism of progressive hearing loss is associated with disruption of the ordered step structure of auditory hair bundles. The screening of deafness genes in deaf family members has expanded the mutational spectrum of EPS8L2, providing a basis for genetic counseling for patients with progressive deafness.

## Author contributions

**Conceptualization:** Hanxiao Gan, Yu Lu, Fengzhu Tang.

**Data curation:** Hanxiao Gan, Huilan Xu, Mingyin Fan, Jie Tang, Jinying Mo.

**Formal analysis:** Yu Lu, Shenhong Qu, Lianli Lu.

**Methodology:** Ruichun Chen.

**Writing – original draft:** Hanxiao Gan.

**Writing – review & editing:** Yu Lu, Shenhong Qu, Fengzhu Tang.
